# Evaluation of Dental Panoramic Radiographs by Artificial Intelligence Compared to Human Reference: A Diagnostic Accuracy Study

**DOI:** 10.3390/jcm13226859

**Published:** 2024-11-14

**Authors:** Natalia Turosz, Kamila Chęcińska, Maciej Chęciński, Marcin Sielski, Maciej Sikora

**Affiliations:** 1 National Medical Institute of the Ministry of Interior and Administration, Wołoska 137 Str., 02-507 Warsaw, Poland; maciej@checinscy.pl (M.C.); marcinsielski@gazeta.pl (M.S.); 2 Department of Maxillofacial Surgery, Hospital of the Ministry of Interior, Wojska Polskiego 51, 25-375 Kielce, Poland; 3 Department of Glass Technology and Amorphous Coatings, Faculty of Materials Science and Ceramics, AGH University of Science and Technology, Mickiewicza 30, 30-059 Kraków, Poland; checinska@agh.edu.pl; 4 Faculty of Applied Sciences, WSB Academy, Cieplaka 1C Str., 41-300 Dabrowa Gornicza, Poland; 5 Institute of Applied Sciences, WSB Merito University in Poznan, Sportowa 29 Str., 41-506 Chorzow, Poland; 6 Department of Oral Surgery, Preventive Medicine Center, Komorowskiego 12, 30-106 Kraków, Poland; 7 Department of Biochemistry and Medical Chemistry, Pomeranian Medical University, Powstańców Wielkopolskich 72, 70-111 Szczecin, Poland

**Keywords:** artificial intelligence, panoramic radiograph, automatic detection, diagnosis

## Abstract

**Background/Objectives:** The role of artificial intelligence (AI) in dentistry is becoming increasingly significant, particularly in diagnosis and treatment planning. This study aimed to assess the sensitivity, specificity, accuracy, and precision of AI-driven software in analyzing dental panoramic radiographs (DPRs) in patients with permanent dentition. **Methods:** Out of 638 DPRs, 600 fulfilled the inclusion criteria. The radiographs were analyzed by AI software and two researchers. The following variables were assessed: (1) missing tooth, (2) root canal filling, (3) endodontic lesion, (4) implant, (5) abutment, (6) pontic, (7) crown, (8) and sound tooth. **Results:** The study revealed very high performance metrics for the AI algorithm in detecting missing teeth, root canal fillings, and implant abutment crowns, all greater than 90%. However, it demonstrated moderate sensitivity and precision in identifying endodontic lesions and the lowest precision (65.30%) in detecting crowns. **Conclusions:** AI software can be a valuable tool in clinical practice for diagnosis and treatment planning but may require additional verification by clinicians, especially for identifying endodontic lesions and crowns. Due to some limitations of the study, further research is recommended.

## 1. Introduction

Radiography plays an important role in diagnostics and treatment planning in modern dentistry. It provides information on anatomy, pathologies, and treatment outcomes. One of the most widely used is a dental panoramic radiograph (DPR). This extraoral technique produces a comprehensive view of dental arches, maxilla, mandible, temporomandibular joints, and partially maxillary sinuses [1]. It is used in many fields of dentistry. In orthodontics, it allows the detection of dental anomalies, evaluation of general dental health, and observation of treatment. DPR also supports maxillofacial surgery in diagnosing dental impactions, dental and mandibular fractures, cysts, and tumors [2]. It involves a small dose of ionizing radiation compared to a dose of cone beam computed tomography (CBCT), which, depending on the scan mode, is about three to six times higher than for DPRs [3,4]. Panoramic radiographs are widely used in epidemiological and screening studies, providing information on the general oral health of large groups of patients [5]. However, they have limitations such as superimposition, distortions, and ghost images [6,7]. Superimposition is the overlapping of structures in the X-ray path and can be caused by tongue rings or developing permanent teeth in the primary dentition [2]. The level of distortion depends on the machine type and the distance between the film and the patient [8]. Ghost images may result from anatomical structures like the cervical spine reflecting over the lower incisor. These artifacts can also be caused by jewelry, including earrings [2]. DPRs lack fine details compared to intraoral radiographs [9]. Therefore, it is not sufficient for examining, for example, proximal dental caries [10]. Similar to other radiographic examinations, DPRs have limited inter- and intra-examiner reliability [11]. Inter-examiner reliability describes the level of agreement among independent examiners when applying a test to the same patient [12]. Intra-examiner reliability is the consistency of an examiner in documenting the same conditions over time [13].

In addition to radiographic imaging, visual-tactile examination, caries detection dye, transillumination, pulp vitality testing, and probing pocket depths are also valuable diagnostic tests in dentistry, crucial for obtaining an accurate diagnosis [14,15]. Early detection of pathological conditions allows for timely interventions and effective dental prevention. Diagnostic tests are also important for monitoring oral health, evaluating treatment outcomes, and assessing risks for dental diseases. They can also be used to screen populations, providing data that inform about the need to implement certain actions and strategies [16].

Artificial intelligence (AI) is the ability of machines to perform tasks traditionally associated with human intelligence, such as learning and problem-solving [17]. It is increasingly applied in dentistry and, according to Thurzo et al., is implemented mainly in radiology and orthodontics [18]. This technology can also be used in detecting root fractures, analyzing the anatomy of the root canal system, and aiding clinicians with working length determination [19]. Machine learning in CAD/CAM software can help manufacture well-made fixed and removable dental restorations [18]. Dental radiography such as CBCT, intraoral, panoramic, and cephalometric radiographs provides large data sets for developing AI-based software [20]. When evaluating DPRs, these algorithms achieve an accuracy of about 90% in detecting caries, osteoporosis, maxillary sinusitis, periodontal bone loss, and teeth identification and numbering on DPRs. Detection of periapical lesions is also characterized by high specificity and sensitivity above 90% [21].

According to the study by Gunec et al., AI can generate faster, more accurate diagnoses than junior dentists with one or two years of experience in detecting periapical lesions [22]. Early detection of pathologies allows rapid implementation of appropriate treatment. AI is also a promising tool in medical screening, as it provides accurate and cost-effective results in less time than traditional methods [23]. Its ability to analyze large data sets facilitates population oral health surveillance [24]. This technology also reduces the risk of human errors resulting from, for example, examiner fatigue [25]. By obtaining a second opinion from the program, dentists can validate their radiograph evaluation and better explain the rationale of the planned treatment.

In the overview of reviews from 2023, twelve systematic reviews regarding the application of AI in the automatic evaluation of DPRs were analyzed [21]. Comparing different algorithms was challenging due to the heterogeneity of the studies, with differences in performance metrics reaching 55%. Possible causes include applying different AI on sample sizes ranging from fifty-five to over a thousand DPRs. The algorithms were used to identify periapical lesions; root canal fillings; and metal- and resin-based restorations, crowns, or implants [26,27,28,29]. Currently, there are very few studies with large sample sizes that assess multiple parameters. This deficiency became the basis for designing and implementing the study reported in this paper.

This study aims to validate AI software as a diagnostic tool and evaluate its sensitivity, specificity, precision, and accuracy in assessing permanent teeth on DPRs. The null hypothesis is stated as follows: “The accuracy of AI software in detecting dental conditions on DPRs is equal to that of human analysis”.

## 2. Materials and Methods

### 2.1. Study Design

This prospective, double-gate study on diagnostic accuracy followed the Standards for Reporting of Diagnostic Accuracy Studies (STARD) and the Checklist for Artificial Intelligence in Medical Imaging (CLAIM) checklists. The research was approved by the Bioethics Committee in Kielce at the Świętokrzyska Chamber of Physicians (approval number: 2.3/2023) and was conducted according to the principles of the Declaration of Helsinki. The National Clinical Trial number assigned to this study is NCT06258798. Patients fulfilling specific eligibility criteria were included. Data was collected in real-time, after the study had begun. The research established two gates with distinct eligibility criteria for AI and human, essential for accurately comparing their diagnostic performances.

### 2.2. Study Population

Patients included in the study were admitted to the radiology department in Kielce, a central European city with about 200,000 inhabitants, and the capital of Świętokrzyskie province in Poland. The eligibility criteria are presented in Table 1. The participant sampling was consecutive. A single reference standard was used for all patients.

### 2.3. Setting

DPRs were taken with the device Carestream CS 9600 (Carestream Health, Rochester, New York, NY, USA) with adjustable exposure conditions set to 60–90 kV and 2–15 mA. Only appropriate quality X-rays, which were performed according to the criteria of the Polish Ministry of Health, were analyzed. The AI algorithm used in the study was integrated with CS Imaging software (version 8, Carestream Dental LLC, Atlanta, GA, USA). After DPRs had been anonymized, the software analyzed the uploaded X-rays (index test). The report included the location of missing teeth, root canal fillings, endodontic lesions, dental crowns, pontics, implants, and implant abutment crowns. Then, radiographs were analyzed by two independent clinical evaluators (N.T. and M.C.) with 4 and 12 years of experience, respectively (reference test). Human analysis was considered the gold reference standard.

### 2.4. Study Size

The required sample size was calculated using the Sample Size Estimation for Diagnostic Accuracy Studies tool [30]. The highest sensitivity or specificity values from the latest publications were considered [21,26,27,29]. The sensitivity value was preferred, and if not available, specificity was chosen. In the recent overview of reviews, the highest sensitivity of detecting missing teeth was 98.1%, while the specificity of identifying endodontic lesions reached 90.24% [21]. According to Kazimierczak et al., the sensitivity of automatic detection of root canal fillings on DPRs achieved 90.7% [26]. Identifying crowns presented a specificity of 95.73% [27]. In the study of Başaran et al., the sensitivity of detecting implants, implant abutment crowns, and pontics was 96.15%, 89.47%, and 77.38%, respectively [29]. We assumed a 5% type I error and a marginal error of 5%.

The actual sample size was 600 DPRs due to the capabilities of researchers—19,200 tests were performed by AI and investigators for each variable. This size was significantly larger than the estimated size for variables: missing (186), root canal filling (2048), endodontic treatment (139), and crown (3470). However, it might be insufficient for the following variables: implant (29,937), implant abutment crown (103,403), and pontic (34,045).

### 2.5. Variables

Table 2 presents the analyzed variables and abbreviations used. The values of variables (1) missing teeth, (2) root canal filling, (3) endodontic lesion, (4) implant, (5) implant abutment crown, (6) pontic crown, (7) dental abutment crown, and (8) sound teeth for analysis performed by AI and investigators were grouped depending on the location of the tooth according to World Dental Federation (FDI) notation, ISO 3950. In this system, each tooth is given a unique two-digit number. The first digit indicates the quadrant of the mouth (the number 1 represents the upper right quadrant, 2—the upper left quadrant, 3—the lower left quadrant, and 4—the lower right quadrant). The second digit indicates the position of the tooth within that quadrant (the number 1,2—incisors, 3—canines, 4,5—premolars, 6,7,8—molars) [31].

### 2.6. Analysis

The acquired data were analyzed in the Microsoft Office program (Microsoft Corporation, Redmond, WA, USA), Google Workspace (version 2024.05.31, Google LLC, Mountain View, CA, USA), and MedCalc software (version 23.0.1; MedCalc Software Ltd., Ostend, Belgium). Based on the analyses performed by AI and investigators, the number of true positive, true negative, false positive, and false negative values were calculated and then used to assess the sensitivity =$\;\frac{TP}{TP+FN}$, specificity =$\;\frac{TN}{TN+FP}$, precision =$\;\frac{TP}{TP+FP}$, and accuracy = $\frac{TP+TN}{TP+FP+TN+FN}$ of the AI program. TP, TN, FP, and FN denote true positives, true negatives, false positives, and false negatives, respectively. To evaluate the results, guideline definitions of “very high”, “high”, “moderate”, and “low” were used (Table A1) [32].

## 3. Results

### 3.1. Participants

**Figure 1:**
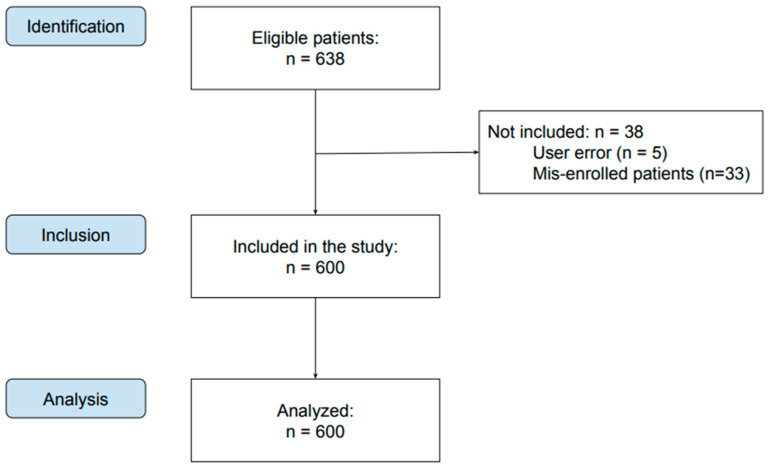
Flow diagram.

The study involved 638 patients. Data on 38 participants were not obtained due to user error (n = 5) or not meeting the eligibility criteria (n = 33). Finally, 600 patients were included in the study—337 females and 263 males (Figure 1). The youngest patient was 11 years old, and the oldest was 81. The average age was 34.78 (SD = 14.48) years.

### 3.2. Test Results

**Figure 2:**
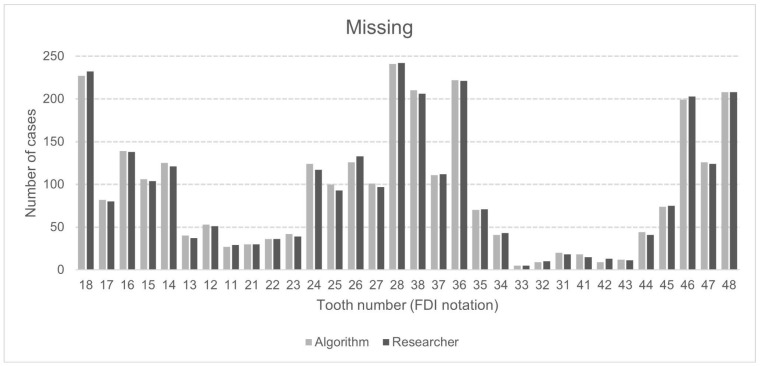
Number of missing teeth detected by AI algorithm and human examiner, with positional detail in upper (18–28) and lower (38–48) dental arch. Position numbering according to ISO 3950:2016 FDI notation (version 2022, FDI World Dental Federation, Geneva, Switzerland) [31].

**Figure 3:**
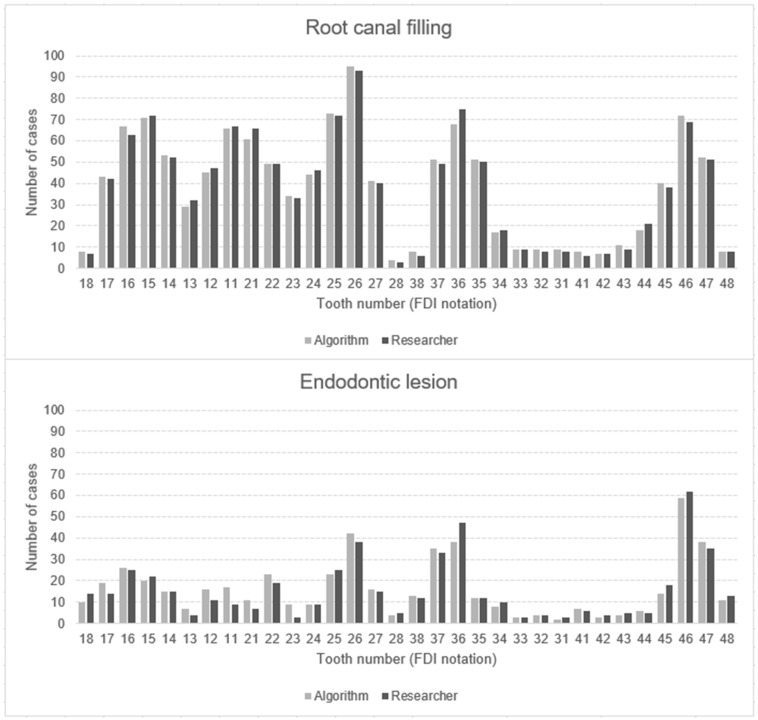
Number of root canal fillings and endodontic lesions detected by AI algorithm and human examiner, with positional detail in upper (18–28) and lower (38–48) dental arch. Position numbering according to ISO 3950:2016 FDI notation (version 2022, FDI World Dental Federation, Geneva, Switzerland) [31].

**Figure 4:**
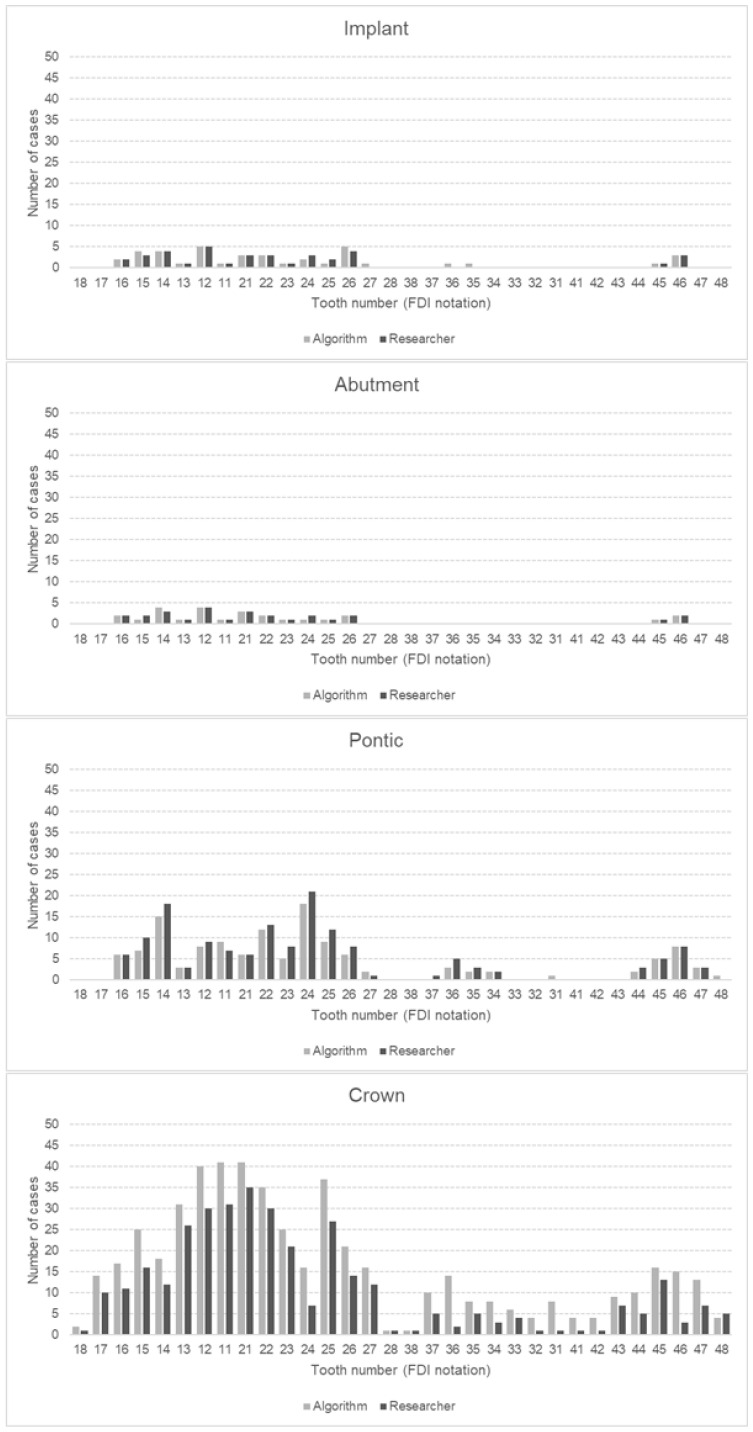
Number of implants, implant abutment crowns, pontic crowns, and dental abutment crowns detected by AI algorithm and human examiner, with positional detail in upper (18–28) and lower (38–48) dental arch. Position numbering according to ISO 3950:2016 FDI notation (version 2022, FDI World Dental Federation, Geneva, Switzerland) [31].

**Figure 5:**
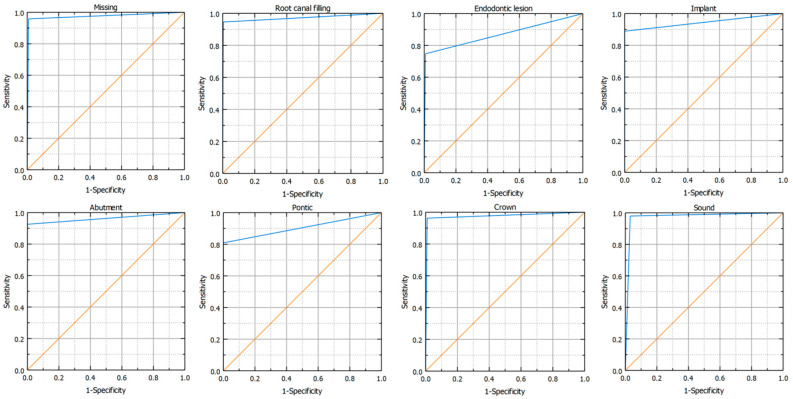
ROC curve representations for the studied variables. The random classifier (diagonal line) is orange, and the ROC curve for the classifier is blue.

In total, both the principal investigator and the AI made 153,600 binary decisions for eight variables and 32 oral positions in 600 DPRs. The researcher had concerns assessing 281 (1.46%) of 19,200 oral locations. The second investigator verified these cases. Table 3 presents the raw results for individual variables and values of sensitivity, specificity, precision, and accuracy. The numbers of teeth with each diagnosis identified by AI and investigators are presented in Figure 2, Figure 3 and Figure 4. Figure 2 illustrates frequency of missing teeth according to their position in both dental arches. The distributions of teeth with endodontic lesions or root canal fillings are presented in Figure 3. Figure 4 provides a comprehensive overview of prosthetic restorations detected on DPRs, showing the comparison in the number of given diagnoses between humans and AI. The receiver operating characteristic curve (ROC) for each variable is presented in Figure 5.

## 4. Discussion

### 4.1. Missing Teeth

Research in recent years has shown a very high effectiveness in identifying missing teeth using AI on panoramic X-rays [33,34,35]. According to Tuzoff et al., the performance of tested CNN-based architecture was comparable to that of experts, which can simplify the filling in of digital dental charts [36]. Vinayahalingam et al., after testing 200 DPRs by the Mask R-CNN architecture, achieved a precision of 99.7% [37]. The results of the present study are also promising. The algorithm detected missing teeth, achieving all analyzed performance metrics above 95%. The sensitivity and specificity were similar to the study from 2024, where CNNs were used for automatic evaluation [38]. The algorithm in the present study marked 22 more teeth as missing than the researchers did. The most common diagnosis differences between AI and clinicians occurred in teeth 24, 25, and 26, while no differences were observed in teeth 21, 22, 33, and 48. Problems with the correct identification of missing teeth for both AI and dentists may occur in the residual dentition when the remaining teeth move toward the edentulous space, leading to their displacement from the proper position (Popov–Godon phenomenon). The algorithm also did not correctly identify teeth when one tooth covered the other, which occurred in the case of crowding or the presence of an impacted tooth. Sometimes, the developing wisdom tooth buds were not detected by the software, either.

### 4.2. Endodontic Lesions and Root Canal Fillings

All analyzed performance metrics for detecting root canal fillings (RCFs) were very high, with values above 94%. These metrics indicate significantly good reliability in making accurate predictions. Other studies also present good results in identifying endodontically treated teeth [26,27]. In a study from 2024, another popular cloud-based AI software, Diagnocat, achieved high accuracy (90.72%) in detecting the probability of fillings [26]. Bonfanti Gris et al., using web-based software Denti.AI, achieved accuracy, specificity, and sensitivity above 90% as well [26,27]. The most common tooth with a RCF was 26. The first molars are the most susceptible to caries because of their early eruption and morphology [39]. Endodontic treatment was least frequently performed in the lower incisors. They are least likely to experience caries because of the large amount of saliva produced by the submandibular salivary glands [40].

**Figure 6:**
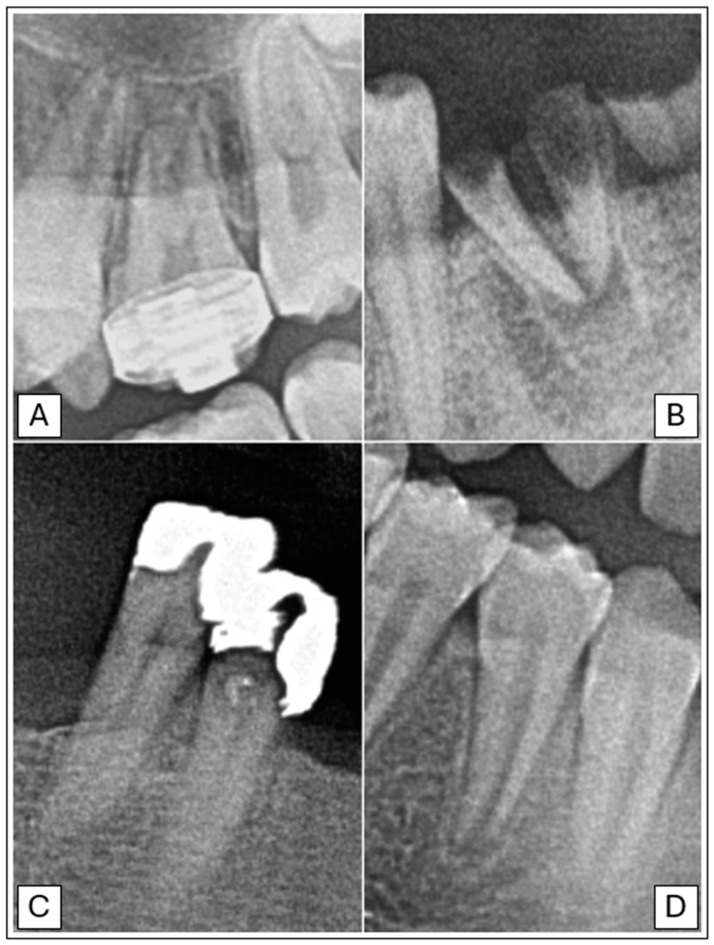
Examples of teeth misdiagnosed by AI software. (**A**) Tooth 26 with an orthodontic band, mistakenly identified as a tooth with a crown. (**B**) Tooth 36, not treated endodontically, incorrectly diagnosed as a tooth with root canal filling. (**C**) Teeth 44 and 45 with extensive, highly radiopaque dental fillings, wrongly identified as teeth with crowns. (**D**) Tooth 44 with incomplete root development, misdiagnosed as a tooth with a periapical lesion.

Lower values of sensitivity (74.75%) and precision (72.47%) were observed in detecting periapical lesions. However, these values are higher than those obtained in other studies, where they achieved 39% and 56%, respectively [11,38]. The reason for the lower value of the indicators may be due to the use of DPR instead of CBCT. Many studies confirmed the higher accuracy of CBCT in detecting periapical lesions, identifying about one-third more periapical lesions [41,42]. A recent study by Kazimierczak et al. has shown that the sensitivity and precision of identifying this pathology were 44.45% and 59.19% higher in the case of AI software analyzing CBCT rather than in DPRs. The specificity and accuracy were high in the case of both these images (above 97%) [43]. There was also a study from 2023 where a model consisting of two convolutional neural networks (CNNs) performed well and detected periapical lesions with accuracy, sensitivity, and specificity above 81% [28]. In the present study, the algorithm sometimes incorrectly identified artifacts, teeth with an undeveloped root apex, and anatomical structures such as the mental foramen or the mandibular canal as periapical lesions (Figure 6). A few times, the software indicated the presence of a lesion in a tooth adjacent to the actual one.

### 4.3. Prosthetics (Crowns, Pontics, Implants, and Implant Abutment Crowns)

The tested algorithm generally performed well in detecting prosthetic elements. All of them achieved very high specificity, above 99%. Moderate precision was observed in identifying crowns. The common problem for the AI was identifying extensive dental fillings, direct veneers, and molar bands as crowns (Figure 6). Nevertheless, the sensitivity, specificity, and accuracy were very high (above 99%). Similar good results in detecting crowns were also obtained in another study where a pre-trained CNN, available online from 2017, was used to analyze 300 DPRs. The sensitivity and precision were 89.53%, while specificity achieved almost 96% [27]. The precision in the study by Altan, where the YOLOv4 model was used, was moderate and reached 74%. However, detecting bridges became more successful, resulting in a precision of 84% [44]. In the present study, all performance metrics for detecting pontics were high, reaching up to 99.95%. A few times the algorithm wrongly indicated teeth as pontics. Another study from 2022 used the AI Model CranioCatch based on the deep CNN method to analyze 1084 DPRs. The detection of pontics was characterized by moderate sensitivity at 77.38% but high precision at 87.83% [29].

The prevalence of implants and implant abutment crowns in the study was low, 0.19% and 0.14%, respectively. Analyzed performance metrics achieved high and very high values. Various studies have also obtained good results for detecting implants. Vinayahalingam et al. achieved a precision of 97.9% [37]. In the study by Başaran et al., the sensitivity and precision were even higher, reaching 96.15% and 92.59%, respectively. However, the algorithm showed slightly lower metrics in detecting implant abutment crowns, with about 3% and 10% decreases [29]. In the present study, all parameters regarding the identification of implant-supported crowns were very high, above 92%. Occasionally, in the present study the algorithm mistakenly identified post and core restorations as implants. 

### 4.4. Limitations

The study only included correctly performed DPRs. Radiographs followed the criteria of the Polish Ministry of Health [45]. However, any qualitative deviation increases the risk of incorrect AI evaluation.

Radiographs of only Caucasian patients were acquired. Additionally, they were performed in one imaging diagnostics center. The lack of diversity due to analyzing a narrow population may limit the generalizability of results. Nevertheless, a typical radiology department was chosen in a medium-sized central European city.

DPRs provide valuable information about teeth and the maxillofacial skeleton. However, they lack details that can be obtained from CBCT or intraoral radiographs. For example, periapical lesions can only be detected on DPRs when the mineral loss of bone reaches 30–50% [46]. Therefore, initial lesions may have been omitted. In the periapical area, condensing osteitis can also be observed, which radiologically appears as a concentric radio-opaque area [47]. Although treatment is only advised if symptoms indicate the need, the algorithm did not include these conditions as periapical lesions. Moreover, superimposition and distortion, which may occur on DPRs, can lead to errors in analysis [2].

In the case of the variables implant, implant abutment crown, and pontic, there is a risk of obtaining an inaccurate result due to a sample size smaller than calculated with the Sample Size Estimation for Diagnostic Accuracy Studies tool [30].

The validity of a study depends on the reliability of the investigator [13]. Human evaluation may be fallible because of examiners’ different experiences or individual perceptions. Fatigue, anxiety, or stress can also affect diagnostic accuracy. More examiners undergoing the same training procedure, called standardization, would objectify the results.

### 4.5. Strengths and Future Perspectives

The strength of this study lies in its large sample size. While most researchers evaluate the reliability of AI algorithms using usually up to 300 radiographs, this study includes more than 600 DPRs, which enhances the validity and generalizability of the findings [25,27,38]. The study delivers a comprehensive comparison of diagnostic accuracy between AI and humans. The results suggest that this technology may serve as a second opinion tool for dental professionals, improving the accuracy of diagnoses and treatments. However, it is necessary to continuously develop the AI algorithms by training them on big data sets and improving the quality of data.

## 5. Conclusions

In summary, the AI algorithm performed well in automatically evaluating DPRs. It achieved very high performance metrics in detecting missing teeth, root canal fillings, and implant abutment crowns, indicating that AI-based software can be a reliable tool for analyzing panoramic radiographs. However, the moderate precision of identifying endodontic lesions and crowns suggest that clinicians should further verify these findings. Due to the limitations of the study, further research is recommended to develop AI algorithms.

## Figures and Tables

**Table 1:** Inclusion and exclusion criteria.

Domain	Criteria for Inclusion	Criteria for Exclusion
Population	Patients of the diagnostic imaging facility in Kielce, Poland	Primary or mixed dentition
Study sample	DPR performed irrespective of the specific indication and assessed by AI	Error resulting in no results or partial results
Control sample	The same DPR assessed by medical professionals	Not applicable
Outcomes	Differences in individual assessments by AI and medical professionals	Not applicable

**Table 2:** Variables.

Abbreviation	Name of Variable	Description
M	Missing	Absence of a natural tooth in a given position of the dental arch
R	Root canal filling	The presence of a filling in at least one root canal
E	Endodontic lesion	Periapical radiological radiolucency suggesting periapical inflammation
I	Implant	Any type of dental implant
A	Abutment	Any superstructure attached to an implant
P	Pontic	A prosthetic crown that replaces a missing tooth in a bridge
C	Crown	A prosthetic crown supported on a tooth
S	Sound	A tooth without the above-mentioned diagnoses

**Table 3:** Outcomes.

	Missing	Root Canal Filling	Endodontic Lesion	Implant	Abutment	Pontic	Crown	Sound
Test outcome negative (index test)	16,223	17,980	18,677	19,162	19,175	19,068	18,687	4890
Actual condition negative (human reference)	16,245	17,984	18,693	19,164	19,173	19,048	18,852	4764
Test outcome positive (index test)	2977	1220	523	38	25	132	513	14310
Actual condition positive (human reference)	2955	1216	507	36	27	152	348	14436
Prevalence in the sample	15.39%	6.33%	2.64%	0.19%	0.14%	0.79%	1.81%	75.19%
True positive results—correctly identified	2832 (14.75%)	1150 (5.99%)	379 (1.97%)	32 (0.17%)	25 (0.13%)	123 (0.64%)	335 (1.74%)	14,143 (73.66%)
True negative results—correctly excluded	16,100 (83.85%)	17,914 (93.30%)	18,549 (96.61%)	19,158 (99.78%)	19,173 (99.86%)	19,039 (99.16%)	18,674 (97.26%)	5197 (27.07%)
False positive results—over diagnosed	145 (0.76%)	70 (0.36%)	144 (0.75%)	6 (0.03%)	0 (0%)	9 (0.05%)	178 (0.93%)	167 (0.87%)
False negative results—misdiagnosed	123 (0.64%)	66 (0.34%)	128 (0.67%)	4 (0.02%)	2 (0.01%)	29 (0.15%)	13 (0.07%)	293 (1.53%)
True positive rate—sensitivity (95% confidence interval in parentheses)	95.84% (95.05–96.53%)	94.57% (93.15–95.78%)	74.75% (70.74–78.48%)	88.89% (73.94–96.89%)	92.59% (75.71–99.09%)	80.92% (73.76–86.83%)	96.26% (93.70–98.00%)	97.97% (97.73–98.19%)
True negative rate—specificity (95% confidence interval in parentheses)	99.11% (98.95–99.25%)	99.61% (99.51–99.70%)	99.23% (99.09–99.35%)	99.97% (99.93–99.99%)	100% (99.98–100.00%)	99.95% (99.91–99.98%)	99.06% (98.91–99.19%)	96.89% (96.39–97.34%)
Positive predictive value—precision (95% confidence interval in parentheses)	95.13% (94.32–95.83%)	94.26% (92.86–95.41%)	72.47% (68.94–75.73%)	84.21% (70.38–92.29%)	100% (86.28–100.00%)	93.18% (87.62–96.35%)	65.30% (61.89–68.57%)	98.83% (98.65–98.99%)
Accuracy (95% confidence interval in parentheses)	98.60% (98.43–98.77%)	99.29% (99.16–99.41%)	98.58% (98.41–98.75%)	99.95% (99.90–99.98%)	99.99% (99.96–100.00%)	99.80% (99.73–99.86%)	99.01% (98.86–99.14%)	97.68% (97.46–97.88%)

## Data Availability

All synthesized data are available in the article. The raw data supporting the conclusions of this article will be made available by the authors on request.

## Appendix A

**Table A1:** Guideline definitions of the levels of performance metrics.

Level	Estimated Performance Metrics
Very high	90–100%
High	80–90%
Moderate	65–80%
Low	below 65%
